# Retraction: Research on the emotional generation mechanism and optimization pathways of traditional village landscapes in northeastern Hubei based on EEG measurements

**DOI:** 10.3389/fpsyg.2026.1933994

**Published:** 2026-07-15

**Authors:** Frontiers Editorial Office

The journal retracts the article cited above.

Following publication, concerns were raised regarding the scientific validity of the article. An investigation was conducted in accordance with Frontiers' policies.

The concerns were deemed to be valid and as a result, the conclusions of the article have been deemed unreliable, and the article has been retracted.

This retraction was approved by the Chief Editors of Psychology and the Chief Executive Editor of Frontiers. The authors do not agree to this retraction.

Frontiers would like to thank Klaus Gramann for contacting the journal regarding the published article.

